# Neuroprotective effects of *Pinus eldarica *in a mouse model of pentylenetetrazole-induced seizures 

**DOI:** 10.22038/AJP.2021.18562

**Published:** 2021

**Authors:** Somaieh Mansouri, Mahmoud Hosseini, Farimah Beheshti, Mohammad-Ali Sobhanifar, Hassan Rakhshandeh, Akbar Anaeigoudari

**Affiliations:** 1 *Pharmacological Research Center of Medicinal Plants, School of Medicine, Mashhad University of Medical Sciences, Mashhad, Iran*; 2 *Department of Anatomy, School of Medicine, North Khorasan University of Medical Sciences, Bojnurd, Iran*; 3 *Division of Neurocognitive Sciences, Psychiatry and Behavioral Sciences Research Center, Mashhad University of Medical Sciences, Mashhad, Iran *; 4 *Applied Biomedical Research Center, Mashhad University of Medical Sciences, Mashhad, Iran *; 5 *Neuroscience Research Center, Torbat Heydariyeh University of Medical Sciences, Torbat Heydariyeh, Iran*; 6 *Department of Physiology, School of Paramedical Sciences, Torbat Heydariyeh University of Medical Sciences, Torbat Heydariyeh, Iran*; 7 *Department of Physiology,* *School of Medicine, Jiroft University of Medical Sciences, Jiroft,* *Iran*

**Keywords:** Pinus eldarica, Pentylenetetrazole, Oxidative stress, Dark neurons

## Abstract

**Objective:**

Oxidative stress has pernicious effects on the brain. *Pinus eldarica* has antioxidant properties. We explored neuroprotective effect of *P. eldarica* against pentylenetetrazole (PTZ)-induced seizures.

**Materials and Methods:**

Male mice (BALB/c) were grouped as control, PTZ, Soxhlet (Sox) 100, Sox 200, Macerated (Mac) 100 and Mac 200 groups. Sox and Mac extracts (100 and 200 mg/kg) were injected during 7 days. Delay in onset of minimal clonic seizure (MCS) and generalized tonic- clonic seizure (GTCS) was measured. Number of dark neurons (DN) and levels of oxidative stress indicators in the hippocampus were evaluated.

**Results:**

Onset of MCS and GTCS was later in groups treated with the extracts than the PTZ group (p<0.01 and p<0.001). Number of DN in the hippocampus in the PTZ group was higher than the control group (p<0.001) while in the extract groups, was lower than the PTZ group (p<0.05, p<0.01 and p<0.001). MDA level was higher whereas total thiol level and activity of SOD and CAT were lower (p<0.001) in the PTZ group than the control group. MDA level in the Sox 100 (p<0.01), Sox 200 (p<0.001) and Mac 200 (p<0.01) groups was less than the PTZ group. Total thiol level in the Sox 200 (p<0.001), SOD in the Sox 100 (p<0.05), Sox 200, and Mac 200 and CAT in the Sox 200 (p<0.001) groups were higher than the PTZ group.

**Conclusion:**

*P. eldarica* prevented neuronal death and reduced seizures caused by PTZ via improving brain oxidative stress.

## Introduction

Oxidative stress resulting from high levels of free radicals has been found as a key factor in neuronal injuries (Xu et al., 2020 ▶). It has been recognized that hydroxyl radical elevates brain neuronal excitability and reduces epileptic seizures threshold via induction of cell membrane dysfunction (Puttachary et al., 2015 ▶). Decreased levels of antioxidant enzymes including superoxide dismutase (SOD) have been also linked to epileptic seizures resulting from oxidative stress (Lee, 2019 ▶). Gamma-aminobutyric acid (GABA) is one of the brain neurotransmitters with antioxidant properties that can protect brain neurons by removing free radicals (Liu et al., 2011 ▶). GABA has two distinct receptors including GABA_A_ and GABA_B_. GABA_A _acts as a chloride ion channel whereas GABA_B_ is coupled to G proteins (Jembrek et al., 2015 ▶). It has been documented that epileptic seizures accompanied by oxidative stress result in the reduction of GABA (Hou, 2011 ▶). The hippocampus is a brain area that is sensitive to oxidative damage and contains GABA receptors (Li et al., 2019 ▶). Oxidative stress has been indicated to enhance the risk of seizure attacks through destruction of GABA-secreting neurons in the dentate gyrus area of hippocampus (Mathern et al., 1999 ▶). There are various substances which are used to induce epileptic seizures in experimental studies (Kumar et al., 2016 ▶). Pentylenetetrazol (PTZ) is one of the well-known drugs frequently employed to trigger epileptic seizures (Asgharzadeh et al., 2020 ▶; Hosseini et al., 2009 ▶; Hosseini et al., 2012 ▶). This GABA_A _receptor blocker has been realized to stimulate the epileptic seizures via disturbing lipid peroxidation, reduction of glutathione and attenuation of SOD and catalase (CAT) activity (Solati et al., 2019 ▶). Oxidative stress status has been shown to occur after seizure attacks induced by PTZ (Karami et al., 2015 ▶; Ebrahimzadeh Bideskan et al., 2015 ▶; Hosseini et al., 2009 ▶). Epileptic seizures induced by PTZ have been reported to be followed by neuronal injury (Homayoun et al., 2020 ▶; Ebrahimzadeh-Bideskan et al., 2018 ▶; Pourzaki et al., 2017 ▶; Seghatoleslam et al., 2016 ▶; Vafaee et al., 2015 ▶). 

*Pinus eldarica *is a species from the family Pinaceae which possesses gray shell, needle-shaped leaves and single or pair cones (Ghaffari et al., 2019 ▶). Presence of polyphenolic commixtures including catechin and taxifolin has been affirmed in extract of this evergreen plant (Sarvmeili et al., 2016 ▶). Some therapeutic effects have been attributed to *P. eldarica* in traditional medicine. The effects of essential oils of *P. eldarica* in alleviation of pain, decrease of inflammatory release and improvement of microbial infections were documented (Iravani and Zolfaghari, 2014 ▶; Ghaffari et al., 2019 ▶). *P. eldarica* has been also reported to cure asthma and relieve skin irritations (Ghadirkhomi et al., 2016 ▶). Decrease in serum level of cholesterol and improvement of atherosclerosis in rabbits were attributed to the nut of *P. eldarica* (Huseini et al., 2015 ▶). The antibacterial effect of *P. eldarica* extract against *Pseudomonas* *aeruginosa* has been also approved (Sadeghi et al., 2016 ▶). In addition, antioxidant and antineoplasmic (Guri et al., 2006 ▶) impact of the extract of needle leaves of *P. eldarica* has been indicated. Previously, we showed that *P. eldarica* has an anticonvulsant effect (Bargi et al., 2017 ▶). Nevertheless, neuroprotective effects of soxhlet and macerated extracts of *P. eldarica* on seizures caused by PTZ were not studied. Therefore, we evaluated the neuroprotective effects of soxhlet (Sox) and macerated (Mac) extracts of *P. eldarica* in a mouse model of PTZ-induced seizures. 

## Materials and Methods

**Preparation of Sox and Mac extracts**

*Pinus eldarica* leaves were mustered from Mashhad city in Khorasan Province, Iran. For preparation of Mac extract, the leaves of the plant were completely comminuted and macerated in ethanol (70%) for 72 hr; this process was repeated three times. Then, solution was filtered through a filter paper. In the last process, the solvent was dissociated by a rotary evaporator. Extract was dried and kept at -20^o^C until injection (Ghadirkhomi et al., 2016 ▶). The yield of the extract was 10% (w/w). For preparation of Sox extract, the leaves of the plant were powdered. Then, extraction was carried out using 70% ethanol in a Soxhlet apparatus (Nowak et al., 2021 ▶). After concentrating, the extract was kept at suitable temperature until required. 

**Animals and groups**

In the current study, 60 male mice (BALB/c) were divided into 6 groups (n=10), as follows: 

1. Control group: The animals of this group were treated by saline. 

2. PTZ group: The mice received 100 mg/kg of PTZ. 

3. Sox 100 group: The animals were treated by 100 mg/kg of Sox extract. 

4. Sox 200 group: The animals received 200 mg/kg of Sox extract. 

5. Mac100 group: In this group, 100 mg/kg of Mac extract was injected. 

6. Mac200 group: The animals received 200 mg/kg of Mac extract. 

Extracts were administered during 7 days. On the 7^th^ day, extracts were infused 30 min before PTZ. Injections were done intraperitoneally. Ethical protocols were executed completely based on the instructions of Ethical Committee of Mashhad University of Medical Sciences (940508). 

**Induction of seizure**

For induction of seizure, 100 mg/kg of PTZ was infused. Then mice were transferred into a plexiglass room and their behavior was observed for 60 min. Severity of seizures was scored based on the following stages: 0, no response; 1, twitch of ears and face; 2, myoclonic seizures without rearing; 3, myoclonic seizures along with rearing; 4, rolling into one side with colic-tonic seizures; and 5, upside down along with generalized clonic-tonic seizures. When an animal showed score 4 and 5, it considered to be fully kindled (Pourmotabbed et al., 2011 ▶). Delay in onset of Minimal Clonic seizer (MCS) and Generalized Tonic-Clonic seizure (GTCS) was computed as indicators of seizure.

**Biochemical assessments**

**Determination of malondialdehyde (MDA) level**

The level of brain MDA was evaluated using a colorimetric method. In this method, reaction of MDA with thiobarbituric acid (TBA) generates a red complex. In this study, 1 ml of brain tissue homogenates was mixed with 2 ml of TBA and trichloroacetic acid (TCA). Then, the prepared mixture was heated in a water bath. After that, the solution reached the room temperature and centrifuged at 1000 rpm. In the last step, the absorbance was measured at 532 nm and MDA concentration was computed by dividing the absorbance by 1.56×10^5^ (Asgharzadeh et al., 2019 ▶). 

**Evaluation of total thiol content**

Determination of the level of brain total thiol was carried out using 2,2′-dinitro-5,5dithiodibenzoic acid (DTNB). Binding the SH group presented in thiol compounds to DTNB produces a color complex with a peak absorbance at 412 nm. In this study, 50 µl of supernatant obtained from brain homogenates was blended with 1 ml of ethylenediaminetetraacetic acid (EDTA). Absorbance measured at 412 nm against Tris-EDTA buffer was considered A1. In the second step, 20 µl of DTNB was added to solution and kept i at room temperature for 15 min. Absorbance of this solution was named A2. In addition, absorbance of DTNB alone was considered absorbance of blank (B). In the last step, the brain level of total thiol was computed using equation shown below (Serejo et al., 2003 ▶): 

Total thiol concentration (mM) = (A2 – A1 – B) ×1.07 / 0.05×13.6

**Assessment of SOD and CAT activity**

SOD activity was determined based on the ability of this enzyme in preventing oxidation of pyrogallol. In this study, 10 µl of supernatant and 20 µl of pyrogallol were mixed. Changes in absorbance were measured by a spectrometer at 570 nm. One unit of enzyme activity was considered the amount of protein which inhibited 50% oxidation of pyrogallol. 

Evaluation of CAT activity was done according to the dissociation of H_2_O_2_ into H_2_O and O_2_ resulting in the reduction of absorbance. Briefly, 1 ml of phosphate buffer, 0.1 ml of samples and 0.4 ml of H_2_O_2_ were mixed. Then, absorbance of the specimens was recorded at 240 nm (Beheshti et al., 2020 ▶).

**Histologic evaluation**

For histological study, fixative solution was administered transcardially. Then, the brains removed from the skull were restricted by paraffin and serial coronal slices (5-µm thickness) were prepared. For staining, the sections were floated in a solution 0.5% of toluidine blue for 30 min at room temperature. Then, the sections were drained using distilled water. In the next step, dehydration of slices was carried out. Finally, slices were photographed under a light microscope (Olympus Corporation) and the number of dark neurons (DN) per unit area of CA_1_, CA_2_, CA_3_ and dentate gyrus (DG) regions of the hippocampus was determined. The average number of DN in each of these areas was computed by the following equation: 

NA=∑Q®af.∑P

"$\sum \overset{\text{®}}{Q}$" shows the sum of counted particles in slices, "a/f" represents the region related with each frame and "ΣP" implies the sum of frame accompanied with point hitting space. 

**Statistical analysis**

Elicited findings are shown as mean±SEM. One way ANOVA followed by Tukey's *post hoc* comparison test was employed for analyzing. Significance level was <0.05. 

## Results

**Effect of Sox and Mac extracts of** ***P. eldarica*** **on PTZ-stimulated seizures**

**Figure 1:**
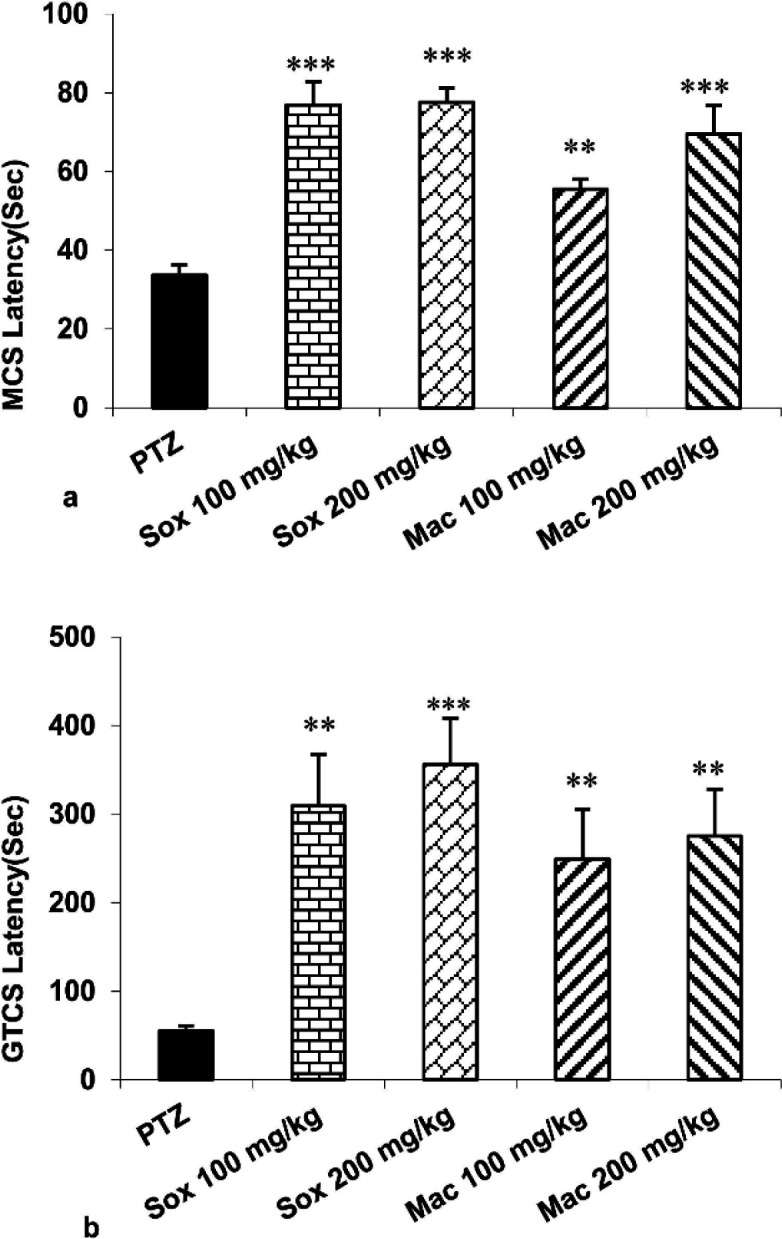
The effects of soxhlet (Sox) and Macerated (Mac) extracts of of *Pinus eldarica* (100 and 200 mg/kg) on the minimal clonic seizures (MCS) (a) and generalized tonic–clonic seizures (GTCS) latencies (b). Data is reported as Mean±SEM. **p<0.01 and ***p<0.001 compared to the pentylenetetrazole (PTZ) group

Intraperitoneal injection of PTZ resulted in the onset and development of MCS and GTCS in mice. Treatment with Sox (100 and 200 mg/kg) and Mac (100 and 200 mg/kg) extracts of *P. eldarica* suspended the onset of MCS and GTCS in mice (p<0.01 and p<0.001). Comparison of delay in onset of MCS and GTCS showed no remarkable difference among Sox100, Sox200, Mac100 and Mac200 groups (Figure 1a and 1b).

**Effect of Sox and Mac extracts of** ***Pinus eldarica*** **on oxidative stress markers in the hippocampal tissue**

**Figure 2:**
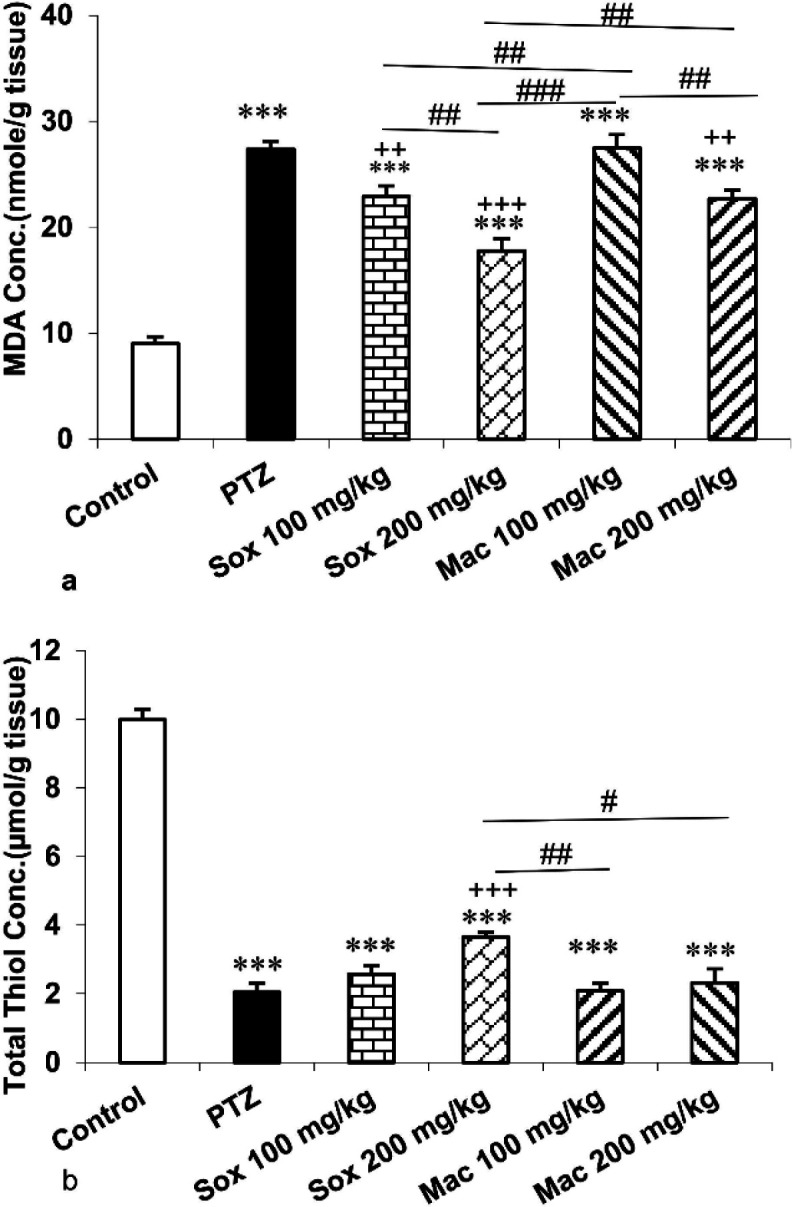
The effects of soxhlet (Sox) and macerated (Mac) extracts of *Pinus eldarica *(100 and 200 mg/kg) on the malondialdehyde (MDA) (a) and thiol (b) concentrations in the hippocampus. Data is reported as Mean±SEM. ***p<0.001 compared to the control group; ^++^p<0.01 and ^+++^p<0.001 compared to the pentylenetetrazole (PTZ) group; and^ #^p<0.05,^ ##^p<0.01 and ^###^p<0.001 compared to other extract treated groups

**Figure 3:**
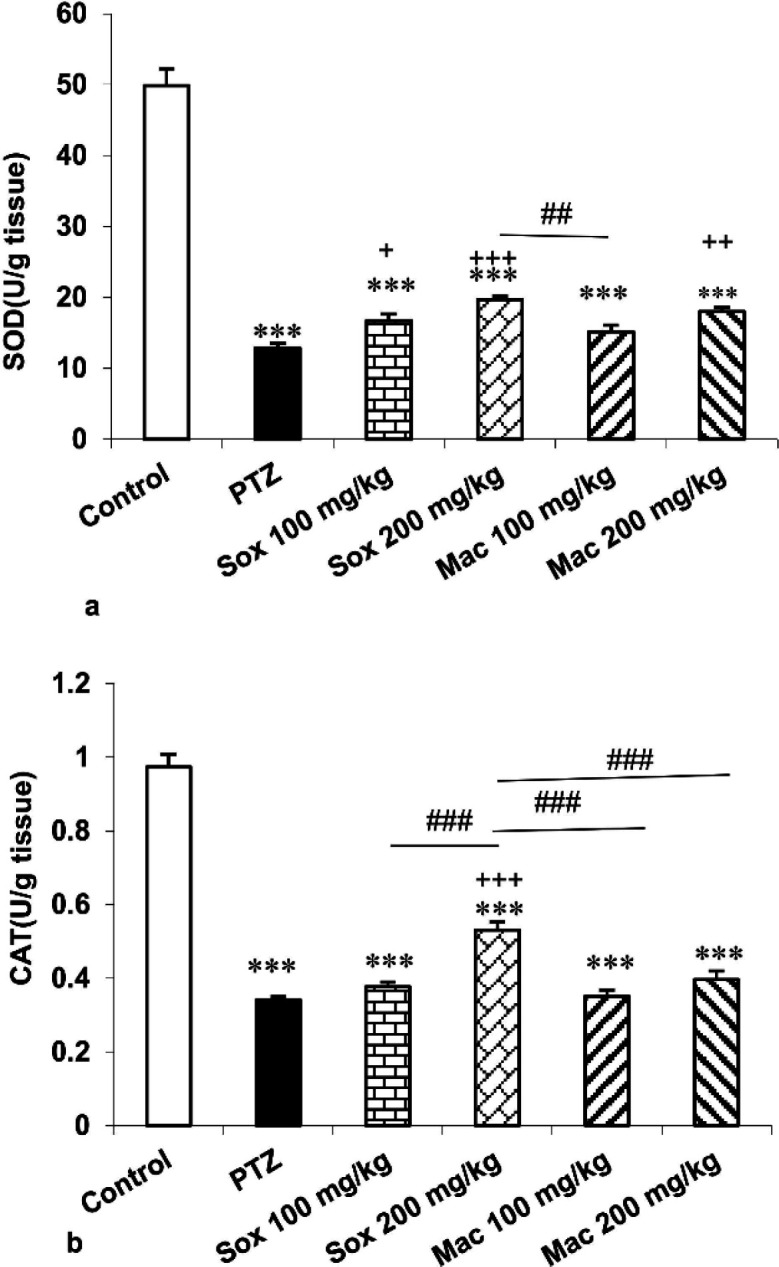
The effects of soxhlet (Sox) and macerated (Mac) extracts of *Pinus eldarica *(100 and 200 mg/kg) on the superoxide dismutase (SOD)(a) and catalase (CAT)(b) activities in the hippocampus. Data is reported as Mean±SEM. ***p<0.001 compared to the control group. ^+^p<0.05,^ ++^p<0.01 and ^+++^p<0.001 compared to the pentylenetetrazole (PTZ) group; and ^ ##^p<0.01 and ^###^p<0.001 compared to other extract treated groups

Biochemical data displayed an enhanced production of MDA, a decreased content of total thiol groups and a weakened activity of SOD and CAT in hippocampal tissue in mice treated with PTZ compared to those of the control group (p<0.001 for all) (Figure 2a, 2b, 3a and 3b). Treatment with Sox (100 and 200 mg/kg) and Mac (200 mg/kg) extracts reversed the effect of PTZ on MDA (p<0.01 and p<0.001). In addition, the MDA level of the hippocampus in the Sox200 group was lower while in the Mac100 group was higher than the Sox100 group (p<0.01 and p<0.001). The MDA level in the hippocampus of both the Mac100 and Mac200 groups was higher than the Sox200 group (p<0.01 and p<0.001). Hippocampal MDA level also in the Mac200 group was lower than the Mac100 group (P<0.01) (Figure 2a). 

Total thiol content in the hippocampus of the Sox200 group was higher than the PTZ group (p<0.001). We did not observe a significant difference in total thiol content between Sox100, Mac100 and Mac200 groups and the PTZ group. Total thiol content in the hippocampus of both the Mac100 and Mac200 groups was lower than the Sox200 group (p<0.01 and p<0.05, respectively). Thiol content in the hippocampus of Sox100, Sox200, Mac100 and Mac200 groups was lower than the control group (p<0.001 for all) (Figure 2b).

SOD activity in the hippocampus of Sox100, Sox200 and Mac200 groups was higher than the PTZ group (p<0.05, p<0.001 and p<0.01, respectively). SOD activity in the hippocampus of Mac100 group was lower than the Sox200 group (p<0.01; Figure 3a). The results also showed that CAT activity of the Sox200 group was higher than the PTZ group (p<0.001). In the hippocampus of Sox200 group, CAT activity was higher than the Sox100 group (p<0.001). In addition, CAT activity in the hippocampus of both the Mac100 and Mac200 groups was lower than the Sox200 group (p<0.001 for both; Figure 3b). Additionally, SOD and CAT activity of the hippocampus in the Sox100, Sox200, Mac100 and Mac200 groups was lower than the control group (p<0.001 for all; Figure 3a and 3b).

Briefly, administration of Sox extract at the dose of 200 mg/kg produced a better effect in reduction of MDA concentration, enhancement of total thiol content and amplification of SOD and CAT activity in hippocampal tissue of mice in comparison with other extracts. 

**Effect of Sox and Mac extracts of** ***P. eldarica*** **on DN production in CA**_1_ **, CA**_2_ **, CA**_3_ **and DG of the hippocampus**

**Figure 4:**
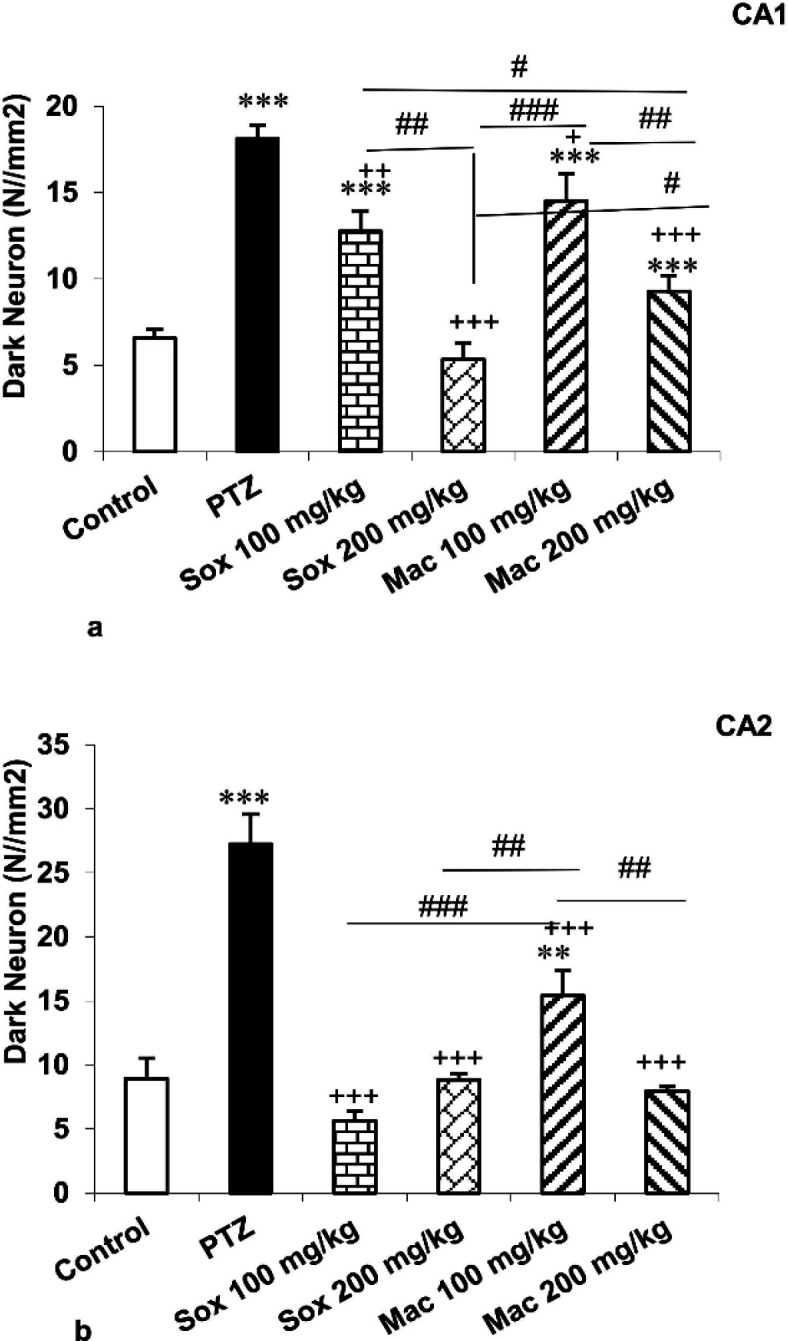
The effects of soxhlet (Sox) and macerated (Mac) extracts of *Pinus eldarica *(100 and 200 mg/kg) on the dark neuron production in CA1 (a) and CA2 (b) areas of the hippocampus. Data is reported as Mean±SEM. **p<0.01 and ***p<0.001 compared to the control group; ^+^p<0.05,^ ++^p<0.01 and ^+++^p<0.001 compared to the pentylenetetrazole (PTZ) group; and^ #^p<0.05,^ ##^p<0.01 and ^###^p<0.001 compared to other extract treated groups

The number of DN in CA_1_, CA_2_, CA_3_ and DG areas of the hippocampus in mice treated by PTZ was higher than the control group (p<0.001). Treatment with both doses of extracts resulted in a significant reduction in the number of these cells in CA_1_, CA_2_ , CA_3_ and DG regions of the hippocampus in the Sox100, Sox200, Mac100 and Mac200 groups compared to the PTZ group (p<0.05 to p<0.001; Figures 4a, 4b, 56a and 6b). Based on the results, the number of DN in CA_1_ of the Sox200 and Mac200 groups was lower than the Sox100 group (p<0.01 and p<0.05, respectively). 

**Figure 5:**
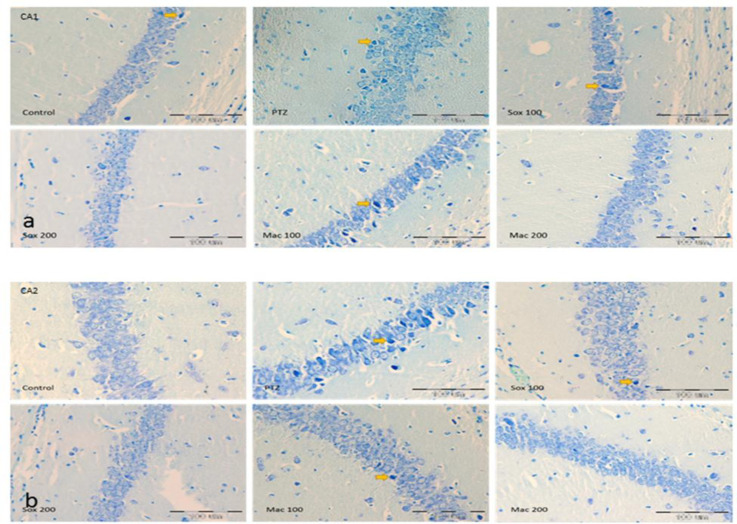
Photomicrograph shows the dark neuron in CA1(a) and CA 2(b) areas of toluidine blue-stained hippocampus in the control, PTZ, Sox 100, Sox 200, Mac 100 and Mac 200 groups, arrow = dark neuron (×200)

Results indicated that the number of DN in CA_1_ region of the hippocampus of Mac100 was higher than the Sox100 group (p<0.05). The number of DN in CA_1_ and CA_2_ regions of the hippocampus of the Mac200 group was also lower than the Mac100 group (p<0.01; Figure 4a and 4b). It was also shown that the DN number in the CA_2_ area of the Mac100 group was higher than the Sox100 and Sox200 groups (p<0.001). The DN number in the Mac200 group was lower than that in the Mac 100 group (p<0.01) (Figure 4b). Photographic pictures of DN in CA_1_, CA_2_, CA_3_ and DG regions of the hippocampus are presented in Figures 5 and 7. 

**Figure 6:**
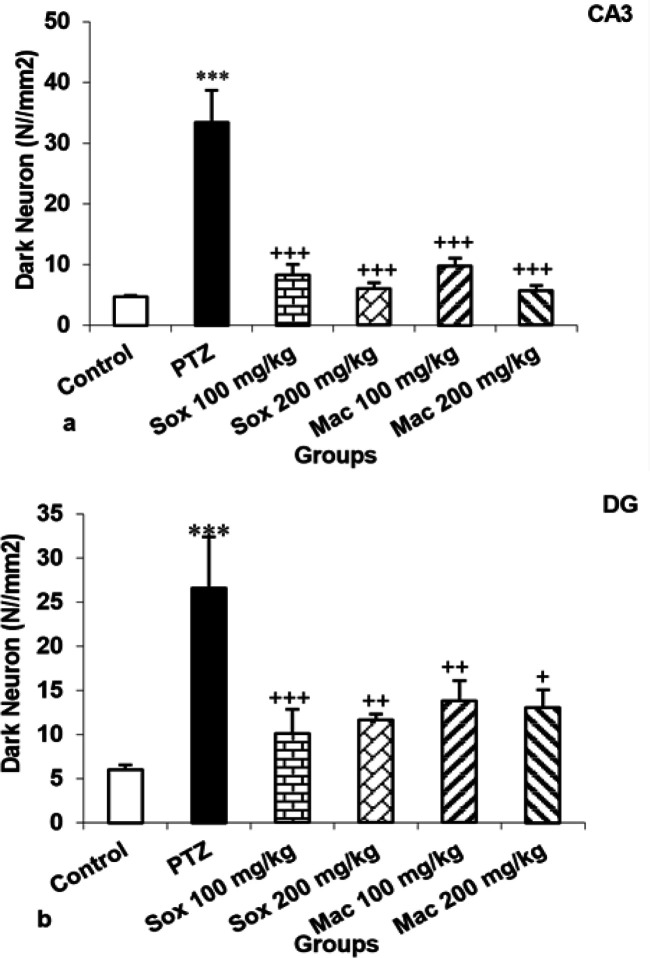
The effects of soxhlet (Sox) and macerated (Mac) extracts of *Pinus eldarica *(100 and 200 mg/kg) on the dark neuron production in CA3 (a) and DG (b) areas of the hippocampus. Data is reported as Mean±SEM. ***p<0.001 compared to the control group, ^+^p<0.05,^ ++^p<0.01 and ^+++^p<0.001 compared to the pentylenetetrazole (PTZ) group

**Figure 7:**
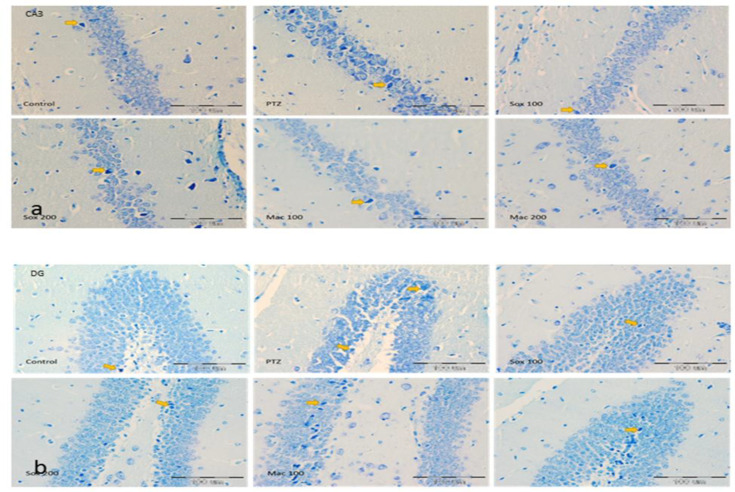
Photomicrograph shows the dark neuron in CA3(a) and DG (b) areas of toluidine blue-stained hippocampus in the control, PTZ, Sox 100, Sox 200, Mac 100 and Mac 200 groups, arrow = dark neuron (×200)

## Discussion

In the present research, PTZ administration triggered epileptic seizures and disturbed brain oxidative status in mice. Pretreatment with Sox and Mac extracts of* P. eldarica *reversed brain damages caused by PTZ via modulating brain oxidative stress. Increased number of injured neurons, reduction in cell size and cellular necrosis in hippocampal area of PTZ-kindled rats were observed (Munguía-Martínez et al., 2019 ▶). DN are known as an important histological index indicating neuronal damage (Jortner, 2006 ▶). These basophilic cells appear following ischemia, oxidative stress and epileptic seizures (Baracskay et al., 2008 ▶; Kherani and Auer, 2008 ▶; Karimzadeh et al., 2012 ▶). In traumatic brain injury, DN have been reported to emerge at a high level in CA_3_ area of the hippocampus and neocortex (Ooigawa et al., 2006 ▶). Enhanced number of DN in DG and CA_1_ areas of the hippocampus was also reported in ovariectomized rats when they were treated by PTZ (Mansouri et al., 2013 ▶). The histological results of the current research also exhibited that the number of DN in CA_1_, CA_2_, CA_3_ and DG regions of the hippocampus of mice was higher in the PTZ group compared with the control group. 

Oxidative and nitrosative stress have been introduced as one of the basic mechanisms in pathogenesis of seizures (Shin et al., 2021 ▶). On the other hand, epileptic seizures can change redox status and disturb the generation and supply of brain energy (Aguiar et al., 2012 ▶). It has been demonstrated that injection of compounds causing epileptic seizures such as kainic acid into CA_3_ region of the hippocampus enhanced free radicals including nitric oxide (NO) and evoked neuronal apoptosis (Chuang et al., 2007 ▶). Based on animal researches, seizures followed by PTZ administration are also linked with remarkable accumulation of MDA, reduction of antioxidant enzymes and decrement of glutathione concentration in multiple organs including the brain, liver and red blood cells (Solati et al., 2019 ▶). In agreement with these reports, the seizures stimulated by PTZ were associated with perturbation in oxidative status in hippocampal tissue of mice in the present research. It was highlighted that MDA level of the hippocampus of mice was higher whereas total thiol concentration and SOD and CAT activity were lower in the PTZ group versus the control group.

*Pinus eldarica* possesses phytochemicals such as polyphenols, tannins and terpenoids (Sarvmeili et al., 2016 ▶). The therapeutic effects of this evergreen plant against constriction of respiratory ducts, injuries of skin and allergic reactions have been confirmed (Huseini et al., 2015 ▶). The hydroalcoholic extract of needles of this plant has been also understood to prolong sleep duration in mice (Forouzanfar et al., 2016 ▶). According to the findings of the current study, Sox and Mac extracts of *P. eldarica* could attenuate the intensity of PTZ-caused epileptic seizures in mice. The latency in onset of MCS and GTCS in mice treated by both of the extracts was longer than those of PTZ group.

Based on scientific reports, the extract of *P. eldarica* possesses compounds with powerful antioxidant properties including β- caryophyllene, β- β-pinene, α-pinene and longifolene (Afsharypuor and San'aty, 2005 ▶). The hydroalcoholic extract of *P. eldarica* has been indicated to protect the human endothelial cells against oxidative damage resulted from H_2_O_2 _(Babaee et al., 2016 ▶). It has been recognized that antioxidant and anti-inflammatory effect of extract of needle part of *P. eldarica* is accompanied by attenuation of cyclooxygenase function and decrease of NO generation (Forouzanfar et al., 2016 ▶; Karonen et al., 2004 ▶). Quercetin that is present in the extract of *P. eldarica *has been also shown to have neuroprotective effects on the central nervous system via improving oxidative stress status (Ishisaka et al., 2011 ▶; Hosseinzadeh et al., 2010 ▶). In the present study, administration of Sox and Mac extracts of *P. eldarica* before PTZ lowered the number of DN in CA_1_, CA_2_ and DG areas of mice hippocampus and modulated PTZ-induced brain oxidative stress. Biochemical results illustrated a decreased accumulation of MDA, an enhanced level of total thiol and an amplified activity of SOD and CAT in the brain of mice treated by the extracts compared to those treated by PTZ. The neuroprotective effect observed in the present study can be attributed to the presence of antioxidant constitutes in Sox and Mac extracts of this plant. It should be kept in mind that besides histological and biochemical tests, doing the behavioral tests can be helpful in further detection of the neuroprotective effects of Sox and Mac extracts of *P. eldarica*. It was a restriction in our work. 

It was also deduced from the results of the current study that Sox extract of *P. eldarica* especially at 200 mg/kg exerted better protective effects against brain damage caused by PTZ than Mac extract. The extract of *P. eldarica* has been shown to have compounds with various polarities which can be purified by different extraction methods (Ghaffari et al., 2019 ▶; Iravani and Zolfaghari, 2014 ▶). Therefore, it seems that the active ingredients responsible for neuroprotective effects in Sox extract of *P. eldarica *are more abundant than its Mac extract. However, this subject needs to be more studied in future. 

In summary, Sox and Mac extracts of *P. eldarica* produced neuroprotective effects against PTZ-induced seizures in mice. This protective property was associated with reduced number of DN and ameliorated brain redox. In addition, data clarified that Sox extract had better improving effects than Mac extract. 
